# Virtual reality paired with nitrous oxide for procedural pain in children: a randomized controlled trial

**DOI:** 10.1097/PR9.0000000000001462

**Published:** 2026-07-22

**Authors:** Maykala J. Williams, Breanne J. Byiers, Nanette Aldahondo, Todd Dalberg, Frank J. Symons, Chantel C. Burkitt

**Affiliations:** a Gillette Children's, Saint Paul, MN, USA; b Department of Educational Psychology, University of Minnesota, Minneapolis, MN, USA

**Keywords:** Virtual reality, Procedural pain, Nitrous oxide, Children, Cerebral palsy, Sedation

## Abstract

**Objective:**

To assess the efficacy and clinical implementation of virtual reality (VR) paired with nitrous oxide for procedural pain management during botulinum toxin (BoNT) injections in children with developmental disabilities.

**Methods:**

A randomized, crossover study was conducted with 31 children with developmental disabilities receiving BoNT injections with nitrous oxide (median age = 10 years, range = 5–17 years; 16 males). Child life specialists facilitated VR during one BoNT appointment and standard of care distraction (eg, music, tablet) at another. Primary outcomes included pain experience assessed using the Faces Pain Scale‐Revised, anxiety, and nitrous oxide concentrations. Secondary outcomes assessed clinical implementation by side effects, preferences, and acceptability.

**Results:**

Children, parents, and child life specialists reported significantly lower pain scores, and nitrous oxide concentrations were reduced in the VR condition compared with standard of care. Virtual reality paired with nitrous oxide was used throughout the procedure for most children (n = 23; 74.2%). Eight children discontinued VR during the procedure (preference [n = 3], mild side effects [n = 5]). The procedure duration did not differ between conditions, and parents reported greater satisfaction with their experience when VR was used.

**Conclusion:**

On average, when VR was used, pain levels were lower while also using lower nitrous oxide concentrations. Implementation of VR into the clinical context, paired with nitrous oxide, appeared beneficial and clinically efficient. Virtual reality in combination with nitrous oxide may provide synergistic effects for procedural sedation resulting in improved patient experiences. Further research is warranted to explore potential effect modifiers for a personalized medicine approach.

## Abstract 2

Supplemental Digital Content is Available in the Text.

Virtual reality paired with nitrous oxide during painful pediatric outpatient procedures reduced pain experience, improved parent satisfaction, and may support reduced nitrous oxide concentrations.

## 1. Introduction

Painful medical procedures can lead to needle phobia, fear of medical settings, and treatment avoidance—factors that often contribute to reduced adherence and poorer health outcomes.^27^ Children with developmental disabilities who often undergo many painful medical procedures during their lifetime may be particularly vulnerable.^8,11^ Exposure to repeated painful events early in life can predispose individuals to perceive future painful experiences as more painful, resulting in psychological sequelae and development of traumatic stress symptoms.^34,35^

Nitrous oxide is often used for procedural sedation during painful medical and dental procedures. In the specialty rehabilitation context, nitrous oxide is often used for pain and anxiety management during botulinum toxin (BoNT) injections.^9,18^ Botulinum toxin injections are a common treatment for the reduction of muscle spasticity, particularly in individuals with cerebral palsy (CP). The procedure involves detecting target muscles with a combination of anatomical knowledge and ultrasound guidance then using a needle to make multiple injections of BoNT; results last a few months before repeating.^6^ Nitrous oxide can be associated with minor side effects include nausea, vomiting, dizziness, hallucinations, and headaches.^2,25^ Despite significant improvements in pain experience with nitrous oxide, BoNT injections remain painful for many children.^39^ Exploring additional nonpharmacological options to reduce anxiety and pain is essential to providing adequate health care and treatment adherence.

Nonpharmacological techniques have been explored in the context of BoNT injections, including biofeedback-assisted relaxation training, distraction therapy, and therapeutic humor.^4,29^ Distraction, especially active distraction, is an established effective method for managing pain and anxiety.^5,17,32^ Virtual reality (VR), defined as a computer-generated, simulated environment experienced through auditory and visual sensory stimuli where 3-dimensional interactions are possible, has become increasingly studied and available for pain and anxiety management during medical procedures.^1,15^ Recent meta-analyses and systematic reviews have shown VR can effectively manage pain and anxiety in pediatric patients during a variety of painful medical procedures as well as needle procedures specifically.^1,12,16,33,36^ The studies excluded patients using inhaled sedation (ie, nitrous oxide) and most excluded patients using other forms of sedation (eg, midazolam). Individuals with developmental disabilities were excluded from most studies, and none of the reviewed studies assessed benefit during BoNT injections. Two studies evaluated the potential of VR to reduce pain and anxiety during BoNT injections.^10,14^ Reduction in discomfort with VR use was reported but participants using sedation were excluded, and the studies lacked proper controls.

It is important to gain a better understanding of the safety and efficacy of VR paired with nitrous oxide for pain management for children with developmental disabilities who undergo painful procedures like BoNT injections routinely. We hypothesized that VR paired with nitrous oxide would reduce the pain experienced during painful BoNT injections. In children with developmental disabilities undergoing BoNT injections with nitrous oxide, the primary aim was to assess the efficacy of VR to reduce pain and anxiety scores, and nitrous oxide concentrations. The secondary aim of this study was to better understand the context for implementation of VR into BoNT clinic.

## 2. Methods

### 2.1. Study design and participants

A randomized, open-label, 2-period, 2-sequence crossover study design was used to assess the efficacy of VR paired with nitrous oxide during BoNT injections (ClinicalTrials.gov registration: Randomized Controlled Trial of Virtual Reality, NCT03521076). After ethical approval by The University of Minnesota Institutional Review Board, children were enrolled by research staff and included if they were scheduled to receive BoNT injections with nitrous oxide at the enrolling tertiary hospital, they and their parents could read and speak English, they were between ages 5 and 17 years, and they did not have a diagnosis of epilepsy, history of motion sickness, presence of ventricular shunt, or require general anesthesia for the procedure. Exclusion criteria were based on safety recommendations following the VR manufacturer's health and safety guidelines for use.

After informed consent and assent (if applicable), participants' next BoNT appointments were assigned to VR for one and standard of care (SoC) for the other in random order using a 1:1 allocation ratio with a randomization table and function built in Research Electronic Data Capture (REDCap).^19,20^ Blinding was not used because of the inability to practically blind participants, parents, or providers.

### 2.2. Standard of care and intervention conditions

Child life specialists (CLS) facilitated distraction support and care for both conditions. Distraction provided as SoC included movies/videos/music on a tablet or phone, small toys, and talking with the child. Distraction methods used in the SoC condition were guided by participant preference and CLS recommendations. Child life specialists were unable to be present for 1 visit of 62 total visits (1.6%; SoC condition) where distraction was provided by nursing staff. The VR used for the study (AppliedVR, SoothVR: California) offered a variety of immersive experiences, including interactive games as well as more passive, guided meditations and scenery tours. Child life specialists and respiratory therapists (RT) were trained in using the VR and had practice fitting the VR with the inhalation mask. Child life specialists supported children to make their own choices for VR experiences to assure age and content-appropriateness as they do for other SoC distraction tools. Children were encouraged to try VR while working with their CLS before their procedure and then to begin VR a few minutes before the start of their injections, and to continue VR use for the entirety of the procedure, if willing and able. Children could remove the headset at any time but remained in the study and were analyzed according to their randomly assigned condition. Botulinum toxin injections were administered by the participant's physical medicine and rehabilitation (PMR) physician. Nitrous oxide concentration levels were managed by a RT. Nitrous oxide concentrations were adjusted per SoC based on each child's response to sedation and the procedure.

### 2.3. Primary efficacy outcomes

Pain was assessed by child self-report, parents report, and CLS report, using the Faces Pain Scale‐Revised (FPS-R).^21^ Faces Pain Scale-Revised scores range from 0 to 10. Parent and child FPS-R scores have previously shown moderate to good correlations.^38^ For both self-report and parental report, FPS-R scores were collected for 3 time points during each treatment session: once before initiation of BoNT injections, and twice after the termination of BoNT injections, with one representing the amount of pain experienced during the procedure, and one representing the amount of pain the individual was experiencing after the procedure had ended. For CLS, FPS-R scores were collected once only, representing the amount of pain experienced during the procedure. Child self-reported pain experienced during injections, using the FPS-R, was considered the primary outcome. Worst pain during the procedure was collected using a visual analogue scale (VAS range 0–100). This was reported once after the termination of injections by self-report, parent report, and CLS report.

Anxiety was assessed by child self-report, parental self-report, and parental proxy-report of the child's anxiety (VAS 0–100). For self-report of anxiety, anxiety scores were collected for 3 time points during each treatment session: once before initiation of BoNT injections, and twice after the termination of BoNT injections, with one representing the amount of anxiety experienced during the procedure, and one representing the amount of anxiety the individual was experiencing after the procedure had ended. For parental self-report of anxiety, parents were asked to report the amount of anxiety they experienced during the procedure, and how much they were experiencing after the termination of the procedure. Parental report of their child's anxiety was collected once before the initiation of the BoTN procedure.

Nitrous oxide concentrations at various points throughout the BoNT procedure were collected from the medical records. The maximum nitrous oxide concentration, concentration at the end of the procedure (ending concentration), and concentration the RT suggested for the next BoNT appointment (recommended next time) were recorded. Per standard care, beginning nitrous oxide concentrations ranged from 40% to 70% and were titrated in 5% increments during the procedure.

### 2.4. Secondary implementation outcomes

To better understand the context for VR implementation, key patient and clinic factors were measured in terms of (1) adverse events or side effects, (2) preference for VR, and (3) acceptability. Specifically, parents, RTs, and CLS were asked to report any side effects of the VR and the duration and severity of any side effects. If VR use was terminated before the end of the procedure, the reason was documented by parents and clinicians. Children were asked to rate their nausea at the end of each visit (VAS range 0–100).

To evaluate interest in and preference for VR, parents and children were asked to report how helpful they expected VR to be (VAS range 0–100; 0 = not at all helpful, 100 = very helpful) before the beginning of the VR visit.

Parents and CLS were asked single-item questions regarding the proportion of time spent thinking about the child's pain (VAS range 0–100; 0 = none of the time, 100 = the whole time). Parents were asked about their satisfaction with pain management (VAS range 0–100; 0 = very unsatisfied, 100 = very satisfied) as well as how their health care experience was that day (VAS range 0–100, 0 = very unpleasant, 100 = very pleasant). Each of these responses were recorded after the termination of the BoTN injections. The duration of the BoNT procedure was collected from the medical record.

### 2.5. Study procedures

Before BoNT injections, participants were given paper questionnaires which included clearly marked participant-report and parent-report questions to be answered before and after the BoNT appointment. Baseline assessments were completed before the child began interacting with the VR device, and later assessments were completed after the VR device had been removed. Demographics, diagnoses, and duration of the medical procedure were collected from the medical record (Table 1).

**Table 1:** Study-wide demographics, where values represent count and frequency for categorical variables and median and interquartile range for continuous variables.

	Total sample N = 31
Sex	
Female	15 (48%)
Male	16 (52%)
Age (y)	10 (5)
Primary diagnosis	
Cerebral palsy (CP)	25 (81%)
Traumatic brain injury	2 (7%)
Spinal cord injury	2 (7%)
Posterior fossa syndrome	1 (3%)
Astrocytoma	1 (3%)
CP type (if applicable)	
Spastic	19 (76%)
Mixed	4 (16%)
Dystonic	1 (4%)
Hypotonic	1 (4%)
Primary diagnosis topography	
Diplegia	9 (29%)
Hemiplegia	14 (45%)
Triplegia	3 (10%)
Quadriplegia	5 (16%)
Gross motor function classification system	
I	15 (48%)
II	11 (36%)
III	4 (13%)
V	1 (3%)
Cognitive status	
Typically developing	24 (77%)
Mild impairment	6 (19%)
Severe cognitive impairments	1 (4%)

### 2.6. Statistical analysis

Descriptive statistics were calculated using medians and interquartile ranges or mean values and SDs for continuous variables, as appropriate based on the skewness of the distributions. Frequencies and percentages were calculated for categorical variables. For comparisons between VR and SoC treatments, generalized linear mixed models were calculated for each outcome using the lme4 package.^3^ Because all outcomes except for nitrous oxide concentrations and procedure duration showed significant rightward skewing, generalized models with Poisson distributions were used. For nitrous oxide concentrations and procedure durations, Gaussian models provided the best model fit according to assessment of model residuals. All models included random effects for participant intercepts and fixed effects for treatment (VR vs SoC). For outcomes with 3 assessment timepoints within each visit, models also included fixed effects for time, plus a time by treatment interaction. The treatment by time interaction was retained in all models because of its theoretical significance, regardless of statistical significance, although both models with and without the interaction term were examined for model fit. Removing the interaction term did not result in any changes to the interpretation of the results. To assess possible session order and carry-over effects, 3 nested models were compared for each outcome, and the one with the best model fit (based on AIC/BIC values) was selected. The base model included only the random and fixed effects noted above. The second model also evaluated simple sequence effects (eg, SoC first vs VR first) by including the main effect of treatment condition during the first study visit. The third model evaluated possible carry-over effects by including a sequence by treatment interaction. Because the primary research question was regarding the differences between treatments across time, pairwise comparisons were calculated to examine the treatment effects for each outcome using the ggeffects package,^13^ regardless of omnibus model results.

For all analyses, the significance threshold was defined as a 2-sided *P*-value ≤ 0.05. Because of the relatively small sample and preliminary nature of the study, no corrections were made for multiple comparisons. A sample size of 34 was calculated to have 80% power to detect a medium (d = 0.5) effect size. A complete data analysis approach was used where those with missing data for a measure were excluded from the analysis for that measure, and those who did not complete both study visits or who did not receive nitrous oxide were excluded from the analysis. For the models for outcomes assessed only once during each visit, participants were excluded if either value was missing. For outcomes collected at multiple timepoints within each study visit, participants were excluded from an analysis if they were missing more than a single value. The number of participants with usable data included for each analysis are reported with the results. An intention-to-treat analysis approach was used for all outcomes. The analyses were conducted using R Studio version 4.4.2 (2024.10.31).

## 3. Results

### 3.1. Study population

**Figure 1.**
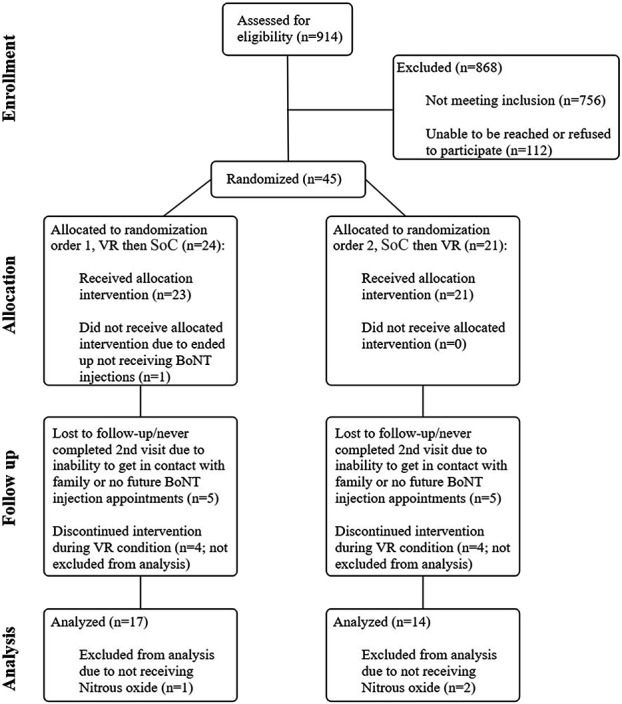
Flowchart of participants through each study phase of this randomized crossover study. Virtual reality (VR), standard of care (SOC), botulinum toxin (BoNT).

A total of 46 children were enrolled, with a total of 31 children (16 male, 15 female) and their parents (24 mothers, 7 fathers) being included in the analysis (Figure 1). Children ranged from 5 to 17 years old, with a median age of 10 years (IQR = 8–14 years). Most children had a primary diagnosis of CP (n = 25, 81%), were Gross Motor Function Classification System (GMFCS) I or II (n = 26, 84%), had typically developing cognitive status (n = 24, 77%), and no relevant comorbid diagnoses (n = 23, 74%; Table 1). Self-report data were unavailable for 4 children because of feeling unwell (eg, nausea), cognitive impairment, or missing data. Enrollment began in September 2018 and concluded in November 2022. Enrollment was paused March 2020 through December 2020 because of the COVID-19 pandemic. The median time between the 2 visits was 7 months 12 days (IQR = 4:24–11:21, min = 3:3, max = 20:2). Because of pause in enrollment or inability to contact family for research, 8 children had BoNT appointments between their research visits. Children received 1 to 13 BoNT injections at each appointment (median = 4, IQR = 2–5). There were no differences in the number of injections between VR and SoC appointments (VR median = 4, IQR = 2–5; SoC median = 4, IQR = 2–5; Mann–Whitney *U* = 445.6, *P* = 0.626). Botulinum toxin appointment duration ranged from 25 to 101 minutes, with a median of 55 (IQR = 44.75–67.0) minutes overall.

### 3.2. Primary efficacy outcomes

#### 3.2.1. Pain scores during procedure

**Figure 2.**
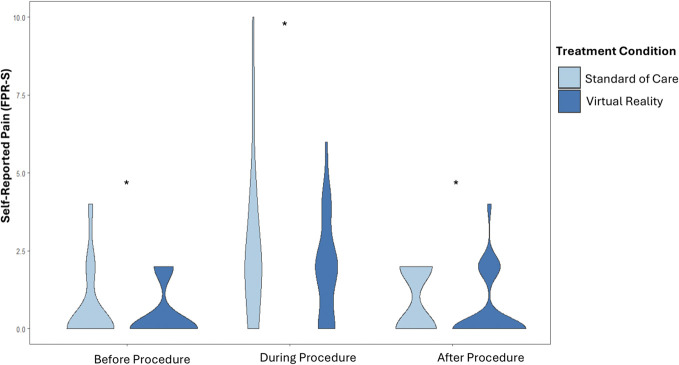
Child self-report of pain before, during, and after the botulinum toxin procedure, assessed using the Faces Pain Scale—Revised (FPS-R). Pain scores were significantly lower across all time points in the virtual reality condition compared with standard of care (*P* < 0.05).

Children's self-reported and parent-reported pain using the FPS-R was significantly lower at all time points during the VR treatment session relative to SoC (Tables 2 and 3; Fig. 2). CLS also reported less pain using FPS-R during VR sessions relative to SoC. For worst pain during the procedure, parent-reported VAS scores did not differ by condition. Although the omnibus model results showed a significant carryover effect for participant-reported VAS pain scores, there were no significant differences between conditions for either sequence group. There was also a significant carry-over effect in the model for CLS-reported VAS pain scores, with results showing no significant difference between conditions among those receiving VR first, but significantly lower scores during VR among those receiving SOC first. Agreement for pain reports across participants, parents, and CLS is reported in Appendix 1, http://links.lww.com/PR9/A418.

**Table 2:** Model comparison metrics for all nested models for each outcome.

Outcome	Base model			With sequence effects (Main effect of condition during first session)			With carry-over effects (Sequence by condition interaction)		
	*n* parameters	AIC	BIC	*n* parameters	AIC	BIC	*n* parameters	AIC	BIC
Feasibility outcomes									
Procedure duration (min)	**4**	**523.0**	**531.5**	5	523.7	534.3	6	525.4	538.1
Parent satisfaction—pain management (VAS)	**3**	**806.0**	**811.9**	4	806.9	814.8	5	808.5	818.5
Parent satisfaction—overall procedure (VAS)	3	723.1	729.1	4	725.0	732.9	**5**	**717.6**	**727.5**
Parent—time thinking about pain management (VAS)	**3**	**688.8**	**694.8**	4	689.9	697.8	5	691.6	701.6
Child life specialist report—time thinking about pain management (VAS)	3	886.9	893.2	4	888.4	896.8	**5**	**851.3**	**861.7**
Primary efficacy outcomes									
Participant—pain ratings (FPR-S)	**7**	**464.7**	**486.3**	8	466.4	491.1	9	468.3	496.1
Parent—pain ratings (FPR-S)	**7**	**445.7**	**467.3**	8	447.7	472.4	9	449.3	477.1
Child life specialist report—pain ratings (FPR-S)	**3**	**208.8**	**215.1**	4	210.7	219.1	5	210.4	220.8
Participant—pain ratings (VAS)	3	778.0	783.9	4	776.9	784.7	**5**	**774.0**	**783.8**
Parent—pain ratings (VAS)	3	791.2	797.2	4	793.0	801.0	5	792.6	802.6
Child life specialist report—pain ratings (VAS)	3	786.2	792.5	4	788.0	796.3	**5**	**717.7**	**728.2**
Secondary efficacy outcomes									
Nitrous concentrations	**6**	**1088.8**	**1108.1**	7	1090.6	1113.2	8	1089.3	1115.2
Participant-reported anxiety (VAS)	7	2693.7	2714.7	8	2695.6	2719.7	**9**	**2679.0**	**2706.1**
Parent proxy-reported anxiety (VAS)	3	802.8	808.9	4	802.6	810.8	**5**	**757.7**	**767.8**
Parent self-reported anxiety (VAS)	5	1446.4	1459.8	6	1447.0	1463.1	**7**	**1415.7**	**1434.5**

Bold font indicates the model with the best fit (ie, lowest AIC and BIC) that was used for subsequent analyses.

AIC, Akaike Information Criteria; BIC, Bayesian information criterion; FPS-R, Faces Pain Scale‐Revised; VAS, visual analogue scale.

**Table 3:** Descriptive statistics and generalized linear mixed model results for parent proxy-reported and child self-reported pain scores.

Outcome measure, data source, and time point	N	Standard of care Median (IQR) EMM (95% CI)	Virtual reality Median (IQR) EMM (95% CI)	Contrast value (95% CI)	*P*
Participant-reported pain (FPS-R; 0–10)*					
Before procedure	27	0 (0–0) 0.51 (0.32, 0.81)	0 (0–0) 0.37 (0.23, 0.59)	−0.14 (−0.28 to 0.00)	0.043†
During procedure		2 (0–4) 2.15 (1.56, 2.96)	2 (0–2) 1.55 (1.10, 2.18)	−0.60 (−0.67 to −0.05)	0.032†
After procedure		0 (0–2) 0.73 (0.48, 1.10)	0 (0–2) 0.53 (0.34, 0.81)	−0.20 (−0.39 to −0.01)	0.039†
Parent-reported pain (FPS-R; 0–10)*					
Before procedure	29	0 (0–1) 0.43 (0.27, 0.70)	0 (0–0) 0.31 (0.19, 0.51)	−0.12 (−0.24 to 0.01)	0.037†
During procedure		2 (2–4) 2.35 (1.71, 3.23)	2 (0–2) 1.69 (1.20, 2.38)	−0.66 (−1.24 to −0.09)	0.024†
After procedure		0 (0–2) 0.65 (0.43, 0.99)	0 (0–1) 0.47 (0.31, 0.72)	−0.18 (−0.35 to 0.02)	0.031†
Child life specialist–reported pain (FPS-R; 0–10)					
During procedure*	30	2 (0–2.5) 1.54 (1.06, 2.22)	1 (0–2) 0.90 (0.59, 1.38)	−0.64 (−1.17 to −0.10)	0.020†
Worst pain during procedure (VAS; 0–100)					
Participant report	26				
Standard of care first	11	17 (0–40) 9.31 (4.09, 21.19)	8 (0–30) 7.43 (3.26, 16.96)	−1.88 (−4.05 to 0.30)	0.901
VR first	15	20 (0–50) 21.83 (11.08, 43.00)	20 (15–44) 22.45 (11.40, 44.22)	0.63 (−2.26 to 3.51)	0.670
Parent report*	27	22.5 (10–56) 21.58 (15.07, 30.90)	15 (6–50) 20.54 (14.34, 29.43)	0.82 (−0.08 to 2.44)	0.322
Child life specialist report	30				
Standard of care first	13	15 (5–50) 13.70 (6.76, 27.8)	9 (0–10) 3.74 (1.81, 7.75)	−9.96 (−17.15 to −2.77)	0.007†
VR first	17	12 (0–30) 12.06 (6.52, 22.29)	15 (1.5–25) 10.43 (5.63, 19.31)	−1.63 (−3.65 to 0.39)	0.113

* That no sequence or carry-over effects were noted for that outcome; values represent values across both sequence groups.

† Statistical significance at alpha = 0.05; EMM, estimated marginal mean; FPS-R, Faces Pain Scale‐revised; VAS, visual analogue scale.

#### 3.2.2. Parental and child anxiety

Children's anxiety scores were not significantly different between conditions, despite a significant carry-over effect in the omnibus model (Tables 2 and 4). For parental proxy-reported anxiety, anxiety scores were lower during the VR session among those whose children received SoC first, whereas anxiety scores were significantly higher during the VR session among those receiving VR first. Results were similar for parental reports of their own anxiety, with the exception of no differences between conditions at the postprocedure time point.

**Table 4:** Descriptive statistics and generalized linear mixed model results for parent proxy-reported and child self-reported anxiety and nitrous oxide concentrations.

Outcome measure, data source, time point, sequence	N	Standard of care Median (IQR) EMM (95% CI)	Virtual reality Median (IQR) EMM (95% CI)	Contrast value (95% CI)	*P*
Participant-reported anxiety	25				
Standard of care first	10				
Before procedure		30 (0–77.5) 15.10 (5.21, 43.78)	10 (0–57.5) 11.67 (4.02, 33.85)	−3.44 (−7.54 to 0.66)	0.100
During procedure		19 (7.5–35) 14.44 (4.98, 41.87)	5 (0–24.5) 8.98 (3.09, 26.08)	−5.46 (−11.50 to 0.58)	0.076
After procedure		0 (0–0) 2.22 (0.75, 6.52)	0 (0–0) 1.29 (0.44, 3.83)	−0.93 (−2.05 to 0.19)	0.105
VR first	15				
Before procedure		10 (2–50) 11.50 (4.82, 27.43)	7 (0–50) 12.42 (5.21, 29.63)	0.93 (−0.70 to 2.55)	0.265
During procedure		0 (0–50) 10.99 (4.61, 26.23)	10 (0–50) 9.56 (4.00, 22.83)	−1.43 (−3.22 to 0.36)	0.117
After procedure		0 (0–10) 1.69 (0.69, 4.10)	0–0) 1.38 (0.56, 3.37)	−0.31 (−0.84 to 0.22)	0.105
Parent proxy-reported anxiety (before procedure only)	28				
Standard of care first	13	22 (0–62.5) 14.15 (6.28, 31.88)	10 (5–55) 10.70 (4.74, 24.14)	−3.45 (−6.74 to −0.17)	0.039*
VR first	15	32 (0–60) 23.63 (11.32, 49.31)	60 (16–76) 33.45 (16.05, 69.70)	9.82 (1.94 to 17.70)	0.015*
Parent self-reported anxiety	27				
Standard of care first	10				
During procedure		27.5 (20–52.5) 26.29 (12.23, 56,49)	12.5 (0–50) 18.42 (8.55, 39.69)	−7.86 (−14.71 to −1.02)	0.024*
After procedure		10 (0–22) 7.23 (33.33, 15.69)	0 (0–12.5) 4.10 (1.88, 8.94)	−3.14 (−5.87 to −0.40)	0.024*
VR first	17				
During procedure		20 (0–30) 11.14 (6.12, 20.30)	20 (7.5–50) 13.71 (7.54, 24.95)	2.57 (0.31 to 4.83)	0.026*
After procedure		0 (0–10) 3.07 (1.66, 5.65)	0 (0–2) 3.05 (1.65, 5.64)	−0.02 (−0.71 to 0.68)	0.963
Nitrous oxide concentrations (30–70)†	31				
Maximum nitrous oxide		60 (55–70) 60.01 (55.75, 64.60)	60 (55–70) 59.54 (55.30, 64.10)	−1.77 (−3.39 to −0.15)	0.033*
Ending nitrous oxide		60 (55–70) 58.26 (54.10, 62.74)	60 (50–70) 56.03 (52.00, 60.38)	−3.55 (−5.17 to −1.93)	<0.001*
Nitrous oxide recommended for next time		60 (55–70) 58.74 (54.55, 63.25)	60 (45–70) 56.03 (52.00, 60.38)	−2.74 (-4.36 to −1.12)	0.002*

* Statistical significance at alpha = 0.05; EMM, estimated marginal mean.

† That no sequence or carry-over effects were noted for that outcome; values represent values across both sequence groups.

#### 3.2.3. Nitrous oxide concentrations

**Figure 3.**
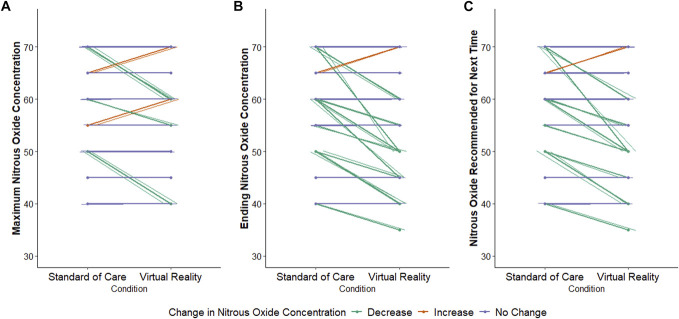
Spaghetti plots depict individual participant scores between conditions for nitrous oxide concentrations with colors indicating decrease (green), increase (orange), or no change (blue) between standard of care and virtual reality. Maximum nitrous oxide concentration indicates the maximum concentration noted in the medical record during the procedure, whereas ending nitrous oxide concentration indicates the concentration noted at the very end of the procedure, and nitrous oxide recommended for next time was noted by the respiratory therapist as the concentration to start at for the next botulinum toxin injection appointment.

On average, children had significantly lower maximum nitrous oxide concentrations for maximum, ending, and recommended concentrations when using VR (Tables 2 and 4; Fig. 3). Seven (23%) children had lower maximum concentrations (5% lower n = 1, 10% lower n = 6), 12 (39%) children had lower ending concentrations (5% lower n = 4, 10% lower n = 6, 15% lower n = 1, 20% lower n = 1), and 11 (35%) children received specific recommendations for lower concentrations for their next procedure after treatment with VR relative to their SOC procedure (5% lower n = 6, 10% lower n = 4, 20% lower n = 1).

### 3.3. Secondary implementation outcomes

#### 3.3.1. Adverse events or side effects

All 31 children successfully used VR paired with nitrous oxide, with no adverse events. Most children (n = 23; 74%) used VR for the entirety of their BoNT procedure. Of the 8 children (26%) who discontinued VR use, 3 discontinued because of preference for using a different distraction strategy (10%). Five children (16%) discontinued VR used because of minor side effects including mild feelings of being overwhelmed or claustrophobic (n = 2), mild dizziness or motion sickness (n = 2), and mild nausea (n = 1). No side effects escalated to adverse events or required any treatment; symptoms resolved by removing the headset. The VAS ratings for nausea after the procedure ranged from 0 to 72, with a median of 0 (IQR = 0–3.75; n = 24). No order effects were identified based on which condition children were randomly assigned first.

#### 3.3.2. Preference for virtual reality

Visual analogue scale scores for how helpful children and parents expected VR to be during the procedure ranged from 0 to 100 for parents (mean = 63.67, SD = 30.04; n = 15) and from 50 to 100 for children (mean = 77.93, SD = 23.24; n = 14).

#### 3.3.3. Acceptability

There were no differences in overall procedure length between the VR and SoC visits (Tables 2 and 5). Parental satisfaction with pain management and proportion of time thinking about their child's pain did not differ between conditions. For parental satisfaction with the overall experience, there was a significant carry-over effect indicating that, although parents whose children received SoC treatment during their first study visit did not report differences in satisfaction between conditions, those whose children received VR during their first study visit reported lower satisfaction during their second (SoC visit). For CLS, there were significant carry-over effects for the proportion of time they reported thinking about pain management during the procedures, such that there were no differences in ratings for participants receiving the SoC treatment during the first session. However, when VR was received during the first session CLS spent more time thinking about pain management during the VR session relative to the SoC session for that child.

**Table 5:** Descriptive statistics and generalized linear mixed model results for selected implementation outcomes.

Outcome measure and data source	N	Standard of care Median (IQR) EMM (95% CI)	Virtual reality Median (IQR) EMM (95% CI)	Contrast value (95% CI)	*P*
Procedure duration					
Medical records*	31	55 (43–66) 55.45 (49.59, 61.31)	55 (45–68) 58.26 (52.40, 64.12)	2.81 (−3.69 to 9.30)	0.390
Satisfaction of pain management during procedure					
Parent report*	27	100 (81.5–100) 83.92 (75.18, 93.68)	100 (90–100) 85.97 (77.03, 95.94)	−2.05 (−6.88 to 2.79)	0.407
Satisfaction with overall experience					
Parent report	27				
Standard of care first	11	90 (73–100) 83.26 (71.31, 97.21)	96 (75–100) 83.62 (71.62, 97.62)	0.36 (−7.19 to 7.90)	0.926
VR first	16	95 (51.75–100) 72.84 (63.96, 82.96)	95.5 (90–100) 88.02 (77.46, 100.00)	15.18 (8.81 to 21.55)	<0.001†
Proportion of time spent thinking about Participant's pain					
Parent report*	27	20 (0–50) 14.94 (8.09, 27.57)	20 (10–50) 15.66 (8.49, 28.89)	−0.72 (−2.20 to 0.76)	0.340
Child life specialist report	30				
Standard of care first	13	10 (0–40) 6.89 (2.82, 16.85)	10 (0–22.5) 6.19 (2.53, 15.15)	−0.70 (−8.36 to 6.97)	0.858
VR first	17	1 (0–17) 6.89 (3.19, 14.87)	15 (5.5, 27.5) 13.24 (6.17, 28.41)	6.35 (1.28 to 11.41)	0.014†

* That no sequence or carry-over effects were noted for that outcome; values represent values across both sequence groups.

† Statistical significance at alpha = 0.05; EMM, estimated marginal mean.

## 4. Discussion

In this randomized, crossover clinical trial, VR effectively reduced the pain experienced during BoNT injections in children with developmental disabilities. Implementation of VR into the clinical context appeared beneficial and nonintrusive. Pairing VR with nitrous oxide was well tolerated. Of the 31 children, no adverse events were experienced and only 5 children discontinued VR due to minor side effects, which resolved quickly. Children, parents, and CLS reported significantly lower pain scores on the FPS-R during BoNT injections when VR was used compared to SoC. On average, children's self-reported pain scores during injections were 28% lower—a clinically meaningful difference.^31^ Parents also rated children's pain during injections 28% lower; whereas CLS rated pain 42% lower when VR was used.

Procedure duration did not differ between VR and SoC conditions, signifying no disruption to clinical flow—important for implementation into fast-paced clinical care. Parents rated their satisfaction 11% higher when VR was used, but only when the VR session occurred first. This finding supports the importance of optimal pain management for improving parent satisfaction, while suggesting that memory of the previous procedure may have affected parents' perceptions. Parents who experienced VR first may have had a new standard for more effective management, influencing their subsequent experience, which was not the case for those who experienced VR second where the distinction may have been less apparent. Child life specialists spent more time thinking about the child's pain during the procedure when VR was used, but only when VR occurred first. This may be because VR was a relatively new modality for CLS. As VR occludes the upper face, CLS may have been exploring additional methods for monitoring signs of pain early in the study. Whereas CLS were likely proficient later in the study when participants were receiving VR second.

Children's anxiety during the procedure did not differ across conditions. Given the significant carry-over effect observed in the model, it is possible that participants experienced more anxiety during the first session (regardless of condition) because of the novelty of study participation, which may have obscured treatment effects. This is further supported by parental reports of anxiety (both for their child and themselves), which was higher in the first condition for both sequence groups. Nitrous oxide concentrations used during BoNT injections were significantly lower when VR was used compared with SoC. Nitrous oxide concentrations at the end of the procedure were, on average, 5% lower; however, concentration reductions up to 20% occurred for some children. Reductions in nitrous oxide concentrations may help alleviate side effects associated with its use such as nausea, vomiting, and hallucinations. To our knowledge, this is the first study examining the efficacy of pairing immersive and interactive 3-dimensional VR with nitrous oxide for outpatient procedural sedation. One previous study paired nitrous oxide with the use of portable video eyewear, a big-screen movie experience within personal eyewear, for outpatient sedation.^37^ Zhang et al. found behavioral reactivity and patient preference were superior for wearing the eyewear in 38 adults undergoing extraction of impacted lower third molars.^37^ The current study extends the work of Zhang et al. as VR headsets, compared with video glasses, are larger and more obtrusive to pair with an inhalation mask and provide an immersive 3-dimensional experience that may be more likely to result in side effects, and our sample extends to children, including those with developmental disabilities.

Other studies that have assessed VR or video glasses in conjunction with inhalation analgesic have assessed use during induction of general anesthesia for surgery for potential reduction of preoperative anxiety.^22,23^ This research is limited but, like our study, found it feasible to use eyewear or a VR headset while simultaneously wearing a sedation inhalation mask, and that level of sedation could still be safely monitored. These findings dispel misconceptions that VR cannot physically fit on the face with the inhalation mask or that sedation levels cannot be monitored with eyes occluded. The pairing of VR and nitrous oxide may provide superior procedural sedation for some children and may reduce the need for strategies like physical holds, which adversely effects the child, parent, and healthcare workers, and can result in negative mental health outcomes and increases the time to accomplish the procedure.^7,26,28^

### 4.1. Limitations

Limitations of the study should be considered. First, of all eligible participants, 29% participated. Study enrollment occurred during the COVID-19 pandemic; many families were unreachable, and families with children with developmental disabilities were disproportionately delaying care and using telemedicine.^24,30^ Second, pain and anxiety measures were based on self-report and proxy report and thus may be subject to response bias. Third, nitrous oxide concentrations were titrated down to some extent in both conditions and the reasons for titration were not specified in the medical record. However, the RCT crossover study design reduces the likelihood of alternative factors explaining the effect between conditions. Fourth, most children in this sample were ambulatory with mild or no cognitive impairment that may limit the generalizability of results to children with more severe developmental disabilities. Because of sample size, we were not able to identify potential effect modifiers that would move the field toward personalized medicine approaches, with known child and clinical factors associated with specific outcomes.

Virtual reality was successfully paired with nitrous oxide sedation to effectively reduce the pain experienced during BoNT injections in children with developmental disabilities. Implementation of VR into BoNT clinic was practical and beneficial. Minor side effects were rare and resolved quickly. Children's self-reported pain scores during injections were 28% lower, on average, when using VR compared with SoC. Parents and CLS also rated pain lower during injections and parents were more satisfied with care when receiving VR. Further research is warranted to explore potential effect modifiers (eg, age, developmental disability severity, comorbidity, prior VR use) to determine factors associated with optimal outcomes and move toward a personalized medicine approach.

## Disclosures

The authors have no conflict of interest to declare.

## Supplemental digital content

Supplemental digital content associated with this article can be found online at http://links.lww.com/PR9/A418.
